# Giardiosis and other enteropathogenic infections: a study on diarrhoeic calves in Southern Germany

**DOI:** 10.1186/1756-0500-7-112

**Published:** 2014-02-26

**Authors:** Julia Gillhuber, David Rügamer, Kurt Pfister, Miriam C Scheuerle

**Affiliations:** 1 Comparative Tropical Medicine and Parasitology, Faculty of Veterinary Medicine, Ludwig-Maximilians-Universität München, Leopoldstr. 5, D-80802 Munich, Germany; 2 Statistical Consulting Unit, Department of Statistics, Ludwig-Maximilians-Universität München, Akademiestr. 1, D-80799 Munich, Germany

**Keywords:** *Giardia*, *Cryptosporidium*, *Eimeria*, *E. coli*, Rotavirus, Coronaviurs, Calf, Diarrhoea, Epidemiology, Prevalence

## Abstract

**Background:**

Diarrhoea induces massive problems in the rearing of calves. The aim of the study was to obtain current data about the frequency of *Giardia* spp., *Cryptosporidium* spp. and *Eimeria* spp. in diarrhoeic calves in Southern Germany with the particular focus on giardiosis.

**Results:**

1564 samples were analysed for the three pathogens using microscopical methods. *Giardia* spp. was detectable in 112/1564 samples (7.2%). The mean age was 46.5 days and the odds of being infected with *Giardia* spp. increased slowly up to 8 times from about 12 days to 30 days of age. There appeared to be no seasonal influence on the frequency of *Giardia* spp. A mono-infection with *Giardia* spp. was diagnosed in 46 calves (2.9%) whereas 15 calves (1.0%) had a mixed-infection with *Cryptosporidium* spp. and 51 calves (3.3%) with *Eimeria* spp. *Cryptosporidium* spp. and *Eimeria* spp. could be detected in 646/1564 samples (41.3%) and 208/1564 samples (13.3%), respectively, with a mean age of 11.3 and 55.0 days, respectively. The odds of being infected with *Cryptosporidium* spp. increased up to 4.5 times until an age of 10 days. After that the odds decreased continuously and was approaching zero at about 30 days. The odds of being infected with *Eimeria* spp. increased continuously up to 30 times from about 20 days to 60 days of age. There appeared to be no significant seasonal influence on the frequency of *Cryptosporidium* spp.; but there was one for *Eimeria* spp.: the odds of being infected with *Eimeria* spp. in March and April decreased by about half and increased up to 2.3 times between July and September.

Additionally, as requested by the veterinarians, 1282 of those samples were analysed for *E. coli*, Rota-, Coronavirus and *Cryptosporidium* spp. using an ELISA. Obtained frequencies for these pathogens were 0.9%, 37.8%, 3.4% and 45.3% with a mean age of 24.8 days, 12.1 days, 9.0 days and 12.1 days, respectively.

**Conclusions:**

The results indicate that in Southern Germany in addition to *Eimeria* spp., *Giardia* spp. seems to play a contributing role in diarrhoea in older calves, whereas *Cryptosporidium* spp. and Rotavirus are mostly relevant in young calves.

## Background

Diarrhoea induces massive problems in the rearing of calves and is often caused by viral, bacterial and parasitic pathogens.

*Giardia* spp., *Cryptosporidium* spp. and *Eimeria* spp. are the most important protozoan parasites causing gastrointestinal problems including diarrhoea in calves.

*Giardia* spp. is commonly found in cattle [1] and although this infection is often subclinical or even asymptomatic, it should be considered as a differential diagnosis in younger calves with acute or chronic diarrhoea, reduced weight gain and ill thrift [1,2]. In this article the taxonomy according to Monis et al. [3] is used. In order to eliminate a taxonomic uncertainty it classifies the Assemblages A-G of the *Giardia duodenalis* morphological group, the causative agents of giardiosis in humans and mammals, as separate species. According to this, there are eleven species within the genus *Giardia, which* can either be distinguished morphologically or genetically. Three of them, the zoonotic species *G. duodenalis* (Assemblages A) and *G. enterica* (Assemblage B) and the livestock-specific species *G. bovis* (Assemblage E) can infect cattle. They are morphologically indistinguishable. *G. duodenalis* and *G. enterica* are also able to infect humans [4] and are therefore of public health significance [5].

Cryptosporidiosis, one of the most important aetiologies of acute diarrhoea, especially in young calves [6], is caused by the intracellular protozoan parasite *Cryptosporidium* spp. Symptoms of this infection can include dehydration, fever, anorexia, weight loss, weakness and progressive loss of condition [7]. Many of the different species and genotypes of the genus *Cryptosporidium*, partly with zoonotic potential, are morphologically indistinguishable [8-10]. One of them is *C. parvum*, a zoonotic genotype often found in young calves and also able to infect humans, hence making the former a source of infection for humans and Cryptosporidiosis a public health issue [2,11]. *C. bovis*, which has the same morphology as *C. parvum,* and *C. andersoni* and *C. ryanae,* which are of another morphological appearance [8,12] are also common causes of infections in cattle [13] but generally they are found in older calves [14-16].

In contrast to these two protozoans with zoonotic potential, *Eimeria* spp. are strictly host specific [17,18]. *E. bovis* and *E. zuernii*, commonly found in calves, are highly pathogenic and often associated with diarrhoea with faeces containing blood, fibrin and intestinal tissue [17,19].

Several studies investigating the impact of the season on a protozoan’s prevalence, had contrasting results with some finding an influence and others not [17,20,21].

*Escherichia coli F5,* Rotavirus and Coronavirus are other important enteropathogens associated with diarrhoea in calves. These three together with *Cryptosporidium* spp. are known to occur in the majority of intestinal infections in calves younger than one month [22].

Differentiation between these viral, bacterial and parasitic agents is only possible by a diagnostic test but not by clinical examination [23]. Since testing under field conditions is not always possible in cases of calf diarrhoea for financial and logistic reasons, it is important for practitioners to know the frequency of the various pathogens in a certain area, the more so as there are no recent data available for Southern Germany.

It is important to emphasize, that management, geographical and climatological parameters and differences in study design, such as the number and age of animals included in the study and the used detection methods can influence the obtained prevalences of pathogens, e.g. *Giardia* spp. [1]. For *Cryptosporidium* spp. it has been shown in a study conducted over 6 years that the prevalences varied markedly between the different years and seasons, although the study design did not change [24]. So when studies differ in one or more of the factors mentioned, the results may vary accordingly.

Thus, the aim of the study was to update the current knowledge of enteropathogenic protozoa in Southern Germany with the focus on the role of *Giardia* spp. as a cause of diarrhoea in calves compared to the other neonatal and post-neonatal pathogens.

## Methods

### Sample collection

Large animal veterinarians in Southern Germany were asked to collect faecal samples from ill patients (diarrhoeic calves; age: < one year) and to send them to our laboratory together with a completed questionnaire. All samples were immediately processed after arrival in the laboratory. Subsequently, the results were sent to the veterinarians and the animals were treated if necessary. Thus, an ethical approval was not necessary, as the samples were gained from patients in order to make a diagnosis.

### Sample analysis

All samples were analysed for *Giardia* spp., *Cryptosporidium* spp. and *Eimeria* spp. using microscopic methods at 200-400x magnifications.

For the detection of *Cryptosporidium* spp. a direct faecal smear of each sample was carbolfuchsin-stained [25], which also allowed to detect *Eimeria-*oocysts. A *Giardia*-infection was diagnosed by using merthiolate iodine formaldehyde concentration (MIFC) method with the addition of Lugol’s solution [26]. Using the MIFC-method also *Cryptosporidium-* and *Eimeria-*oocysts were detectable.

On request of the veterinarians the majority of the samples (1282/1564) were also analysed for *E. coli*, Rota-, Coronavirus and *Cryptosporidium* spp. using the Bio-X Easy-Digest-ELISA (Bio-X Diagnostics S.P.R.L.) in order to increase the diagnostic spectrum of pathogens. This commercial ELISA kit was performed according to the manufacturer’s instructions.

As all samples were processed within the scope of the routine diagnostic in our Quality management certified laboratory, they were initially only analysed by the methods, routinely used there. In order to obtain current data about the frequency of the different *Giardia* species in calves in Southern Germany most of the *Giardia*-positive samples of the present study were genotyped within the scope of another investigation along with further *Giardia*-positive samples of diarrhoeic and healthy calves [27]. Further investigations like quantifying the number of cysts and oocysts, respectively, differentiating *Eimeria*-oocysts and genotyping *Cryptosporidium* spp. by PCR were not performed because of logistic, personnel and financial reasons.

### Statistical analysis

Statistical analysis was run using Microsoft-Excel-2010 software, PASW Statistics 18 (Predictive Analysis SoftWare – SPSS Inc.) and R [28].

The *t*-test for independent samples and an ANOVA (analysis of variance) were performed to investigate the differences in the mean age of the sampled animals in the single months.

For each disease (binary response: yes/no) a generalized additive logistic model was fitted using the statistical program R and the package mgcv [29,30]. The models try to explain the relative risk of infection depending on the age of the calves and the time of the year. Therefore the age of the calves is fitted by using thin plate regression splines with quantile-based knots and the time of the year by using cyclic penalized B-splines [31] and twelve equidistant knots for each month. Because of limited samples of calves > 80 days these models were calculated just using the dates of calves ≤ 80 days. For easier interpretation and visualization on the proportion-scale of infection the smooth curves were transformed. The obtained figures show the multiplicative effect of age respectively the season on the relative risk of infection for a given value of the other covariate.

For all analysis, a P-value of < 0.01 was considered to be significant.

## Results

### Samples

**Figure 1:**
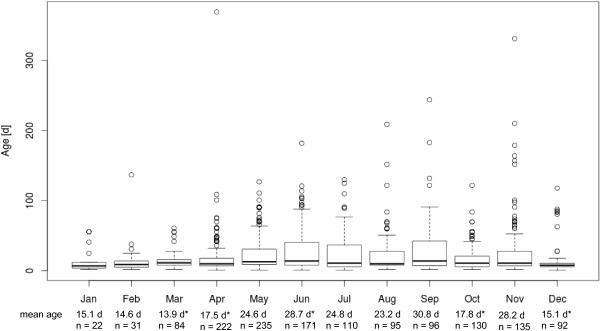
**Age composition and mean age of the sampled animals in the single months (n = 1423).** A significant difference of the mean age of one month in relation to the mean age of the other eleven months (calculated with a *t*-test for independent samples) is marked with *.

From January 1st – December 31st 2012 1564 faecal samples from calves (1–369 days old) were processed. The mean age of the sampled calves was 22.4 days (median = 11 d, n = 1423). The result of the ANOVA shows, that there was a significant difference in the mean age of the sampled animals, sent in in the single months. Furthermore the boxplots, presenting the age composition in the single months, and the results of the *t*-test show in detail, in which months the calves were on average (significantly) younger or older compared with the remaining months (Figure 1).

### Results of the direct detection methods

In 896/1564 examined samples (57.3%) at least one parasite was detectable by microscopical methods.

**Figure 2:**
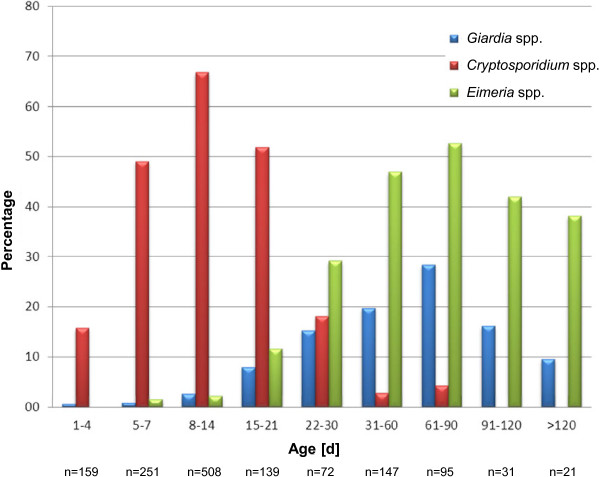
**Age dependent frequencies of cyst/oocysts from *Giardia *** **spp., *Cryptosporidium *** **spp. and *Eimeria *** **spp. (n = 1423).**

**Figure 3:**
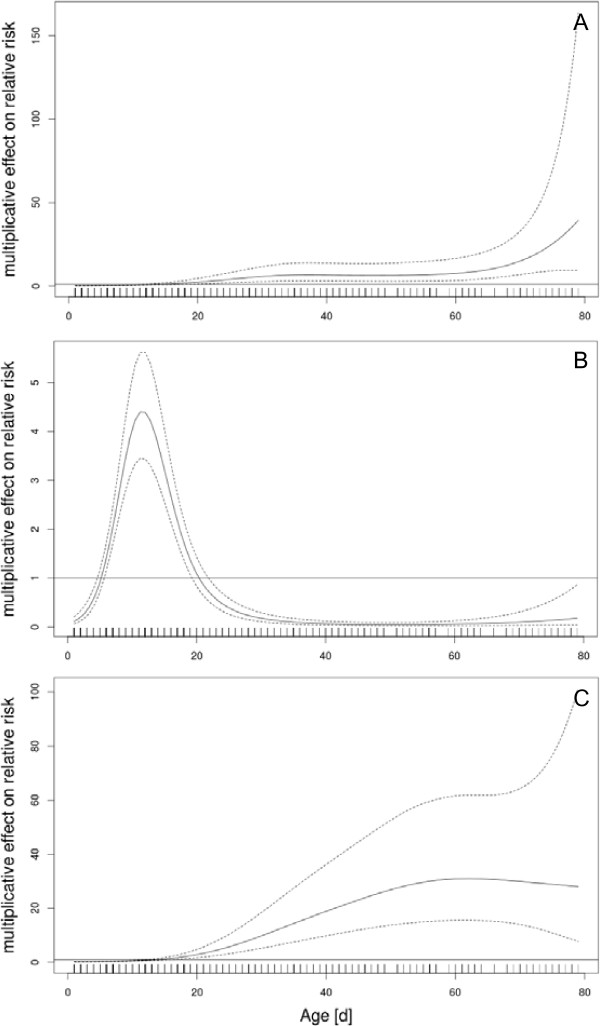
**Odds of infection at different age (n = 1350).** Straight line: level with no influence; −−−: 99% confidence interval. **A**: *Giardia* spp.; **B**: *Cryptosporidium* spp.; **C**: *Eimeria* spp.

**Figure 4:**
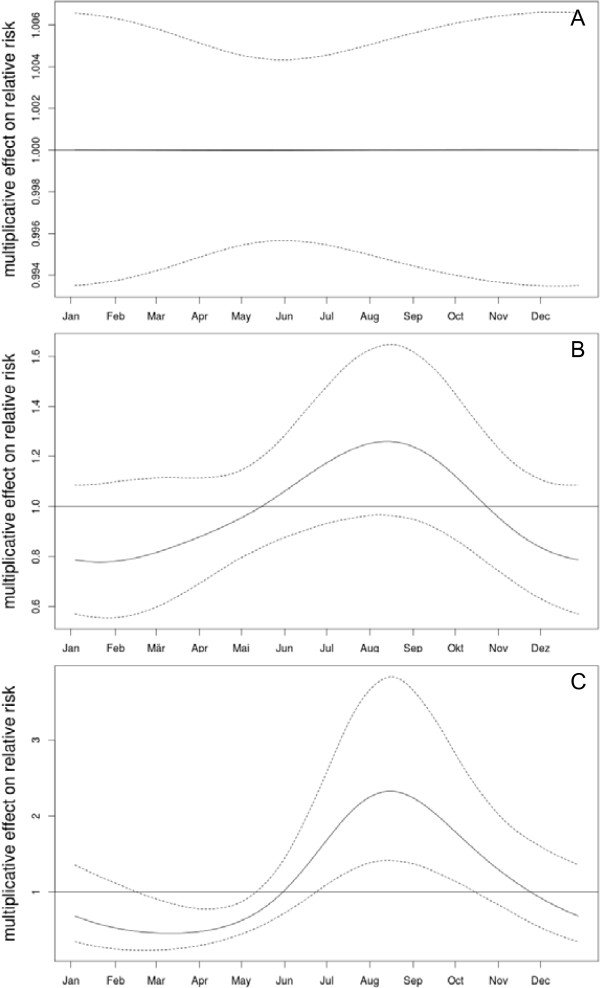
**Odds of infection in the different months (n = 1350).** Straight line: level with no influence; ---: 99% confidence interval. **A**: *Giardia* spp.; **B**: *Cryptosporidium* spp.; **C**: *Eimeria* spp.

*Giardia-*cysts were detected in 112/1564 examined samples (7.2%). The *Giardia*-positive calves were between 3 and 130 days old (mean = 46.5 d, median = 42 d, n = 101) with the highest rate of cyst excretion (28.4%) in 61–90 days old animals (Figure 2). It is significant, that the age had a nonlinear influence on the probability of being infected with *Giardia* spp. and the odds of being infected with *Giardia* spp. increased slowly up to 8 times from about 12 days to 30 days (Figure 3). There was no seasonal influence on the odds of being infected with *Giardia* spp. (Figure 4). A mono-infection with *Giardia* spp. was diagnosed in 46 calves (2.9%) whereas a mixed-infection was found in 15 samples (1.0%) for *Cryptosporidium* spp. and in 51 samples (3.3%) for *Eimeria* spp. (Table 1).

**Table 1:** **Number and percentage of calves with a mono-/mixed-infection with *Giardia *** **spp., *Cryptosporidium *** **spp. and *Eimeria *** **spp.**

**Parasite(s) detected**	**Calves (n = 1564)**	
	**Number**	**%**
*Giardia* spp. only	46	2.94
*Cryptosporidium* spp. only	627	40.09
*Eimeria* spp. only	153	9.78
*Giardia* spp. + *Eimeria* spp.	51	3.26
*Giardia* spp. + *Cryptosporidium* spp.	15	0.96
*Cryptosporidium* spp. + *Eimeria* spp.	4	0.26
None	668	42.71

(Results of the microscopic examination).

*Cryptosporidium*-oocysts were detected in 646/1564 examined samples (41.3%). They had the size and morphology of *C. parvum*. The *Cryptosporidium*-positive calves were between 2 and 77 days old (mean = 11.3 d, median = 10 d, n = 580) with the highest rate of oocysts excretion (66.7%) in 8–14 days old calves (Figure 2). It is significant, that the age had a nonlinear influence on the probability of being infected with *Cryptosporidium* spp. and the odds of being infected with *Cryptosporidium* spp. increased up to 4.5 times until an age of 10 days. After that the odds decreased continuously and was approaching zero at about 30 days (Figure 3). There was no significant seasonal influence on the frequency of *Cryptosporidium* spp. (Figure 4).

*Eimeria*-oocysts were detected in 208/1564 examined samples (13.3%). The *Eimeria*-positive calves were between 5 and 331 days old (mean = 55.0 d, median = 49 d, n = 192) with the highest rate of oocysts excretion (52.6%) in 61–90 days old calves (Figure 2). It is significant, that the age had a nonlinear influence on the probability of being infected with *Eimeria* spp. and the odds of being infected with *Eimeria* spp. increased continuously up to 30 times from about 20 days to 60 days (Figure 3). There was a significant seasonal influence on the frequency of *Eimeria* spp. (Figure 4): the odds of being infected with *Eimeria* spp. in March and April decreased by about half and increased up to 2.3 times between July and September.

The distribution of mono- and mixed-infections is presented in Table 1.

### Results of the ELISA

*E. coli* was detected in 12 (0.9%), Rotavirus in 485 (37.8%), Coronavirus in 43 (3.4%) and *Cryptosporidium* spp. in 581 (45.3%) faecal samples of the part analysed using ELISA (n = 1282). The mean age of the positive tested calves was 24.8 days (n = 12), 12.1 days (n = 443), 9.0 days (n = 39) and 12.1 days (n = 532), respectively. In 612/1282 samples only one pathogen was found (47.7%). Two and three pathogens were found in 250/1282 (19.5%) and 3/1282 (0.2%) calves respectively (Table 2).

**Table 2:** **Number and percentage of calves with a mono-/mixed-infection with *E. coli, *** **Rota-, Coronavirus and *Cryptosporidium *** **spp.**

**Enteropathogen(s) detected**	**Calves (n = 1282)**	
	**Number**	**%**
*E. coli* only	9	0.70
Rotavirus only	245	19.11
Coronavirus only	11	0.86
*Cryptosporidium* spp. only	347	27.07
*E. coli* + Rotavirus	1	0.08
*E. coli* + *Cryptosporidium* spp.	1	0.08
Rotavirus + Coronavirus	18	1.40
Rotavirus + *Cryptosporidium* spp.	218	17.00
Coronavirus + *Cryptosporidium* spp.	12	0.94
*E. coli* + Rotavirus + *Cryptosporidium* spp.	1	0.08
Rotavirus + Coronavirus + *Cryptosporidium* spp.	2	0.16
None	417	32.53

(Results of ELISA-screening).

In the 1282 samples, examined with the microscope and the ELISA, 522 were positive for *Cryptosporidium* spp. in both methods, 59 only in the ELISA and 29 only in the microscopical examination. Thus, of the 610/1282 samples positive for *Cryptosporidium* spp. in the ELISA or the microscopical examination, 95.3% (581/610) could be detected by the ELISA and 90.3% (551/610) by the microscopical examination.

## Discussion

The study reveals that *Cryptosporidium* spp. and Rotavirus are the most prevalent pathogens in diarrhoeic calves up to one year old in Southern Germany, followed by *Eimeria* spp. and *Giardia* spp. Coronavirus and *E. coli* were diagnosed comparatively infrequently.

The detection rate of 7.2% of *Giardia* spp. is much lower compared to the obtained prevalences in many former studies; although prevalences of this pathogen in general are differing markedly as shown previously [1,20,32]. In a recent European study using a commercially available monoclonal antibody-based ELISA, 51.2% of 2–16 weeks old calves in the area of Berlin/Germany were infected with *Giardia* spp. [33]. In the present study the highest detection rate of *Giardia* spp. was in calves between 61 and 90 days of age with 28.4%. This is in line with other studies, which also revealed highest prevalence in about 3 months old calves [20,34]. By contrast other authors reported highest prevalence in 4–7 and 4 week old calves, respectively [35,36], whereas in Huetink et al. [37] it was highest in 4–5 month old animals. An impact of season on the probability of a giardiosis could not be shown. As discussed by Hamnes et al. [20], there are studies, showing seasonal variations in the prevalence of *Giardia* spp., and also other studies, not having found a seasonal impact on the prevalence. The role of giardiosis as a cause of diarrhoea in ruminants is still unclear [8,13]. In former studies *Giardia* spp. is thought to be the reason for diarrhoea and ill thrift in calves [38,39] whereas in others no association between diarrhoea and *Giardia*-infection could be demonstrated [37,40,41]. Giardiosis in ruminants is often asymptomatic. It is unclear, when clinical symptoms appear [8], as this is influenced by many factors like species/breed of host, species of *Giardia*, age, immune competence, frequency of infection, nutrition and concurrent infections [4]. However, also the role of an asymptomatic infection as a cause of production loss in calves is not yet appropriately investigated. Olson et al. [42] showed an association between a *Giardia*-infection and a reduced rate of weight gain, an impaired feed efficiency and a decreased carcass weight in experimentally infected lambs. On the contrary no significant difference in the average weight gain, the feed efficiency and the dry matter intake between *Giardia* spp. infected and non-infected steers could be found by Ralston et al. [43]. The present results indicate that *Giardia* spp., being the only pathogen found in some samples, may contribute to diarrhoea in older calves. In a former study, having examined 20 calves regularly from birth until 4 month of age, an association of giardiosis with diarrhoea was shown, since in a few cases no additional pathogen could be found at the time of a diarrhoea episode [38]. However, these calves had also been diagnosed *Cryptosporidium*-positive at some time during the study, with having found *Cryptosporidium*-oocysts on average earlier than *Giardia*-cysts. So it is possible that a former infection, in this case with *Cryptosporidium* spp., may favour the pathogenic potential of *Giardia* spp. Hence, further investigations are necessary with regard to *Giardia* spp. as cause of diarrhoea in calves and to production loss in asymptomatic calves [8]. In order to obtain current data about the frequency of the different *Giardia* species in calves in Southern Germany and to evaluate a species-specific pathogenicity, a further study was undertaken, including among others most of the *Giardia*-positive samples of the present study [27]: *G. bovis* has been identified in 91.8%, *G. duodenalis* in 7.3% and a mixed template of *G. duodenalis* and *G. bovis* in 0.9% of the PCR-positive samples. This showed that although the livestock-specific species *G. bovis* has been diagnosed most frequently, the potential zoonotic species *G. duodenalis* is also present in calves in Southern Germany and thus might be a risk for animal handlers. Regarding a species-specific pathogenicity the results indicated that the livestock-specific species *G. bovis* might contribute to diarrhoea in calves, as it was the only pathogen found in a proportion of the samples from diarrhoeic calves, whereas *G. duodenalis* was only found in mixed infections with *Cryptosporidium* spp. or *Eimeria* spp.

The highest detection rate was found for *Cryptosporidium* spp. regardless of the examination method. Because of the low age of nearly all calves with a *Cryptosporidium*-infection in this study it is assumed, that almost all of them were infected with *C. parvum*, the species most often found in young calves, although *C. bovis* cannot be completely excluded. In previous studies in Germany the frequencies for *C. parvum* varied between 21.5% and 44.0% and were thus lower than in the present study [44,45]. As reviewed by Hamnes et al. [20] the prevalence of *Cryptosporidium* spp. in studies around the world varies from 6.2 to close to 100%. The age related frequencies in the present study, with the highest detection rate (66.7%) in calves between 8 and 14 days coincide with the results of former studies [14,46]. Not finding a seasonal influence on the risk of being infected with *Cryptosporidium* spp. is in line with the results of some former studies, whereas others have found such an influence [discussed by [20].

*Eimeria*-oocysts were detected in 13.3% of the samples. Compared with other studies in Germany and Austria with prevalences of 59.4% and 83.7%, respectively, this frequency is quite low [19,47]. In these two studies *E. bovis* and *E. zuernii* were the most frequent species found. As the species-differentiation was not performed in this study, we can only assume, that the majority of the *Eimeria*-oocysts of the diarrhoeic animals here probably also belong to these two high pathogenic species [17,19]. In this study the age dependent frequency was highest in calves between 61–90 days of age. This is similar to the results of Lentze et al. [48], who found a significantly increasing risk of an *Eimeria*-infection until 3 months of age. In another investigation the highest prevalence was in animals between 3 and 12 months [49]. An association between season and frequency of *Eimeria* spp. was found in this study, with the highest odds of an Eimeriosis between July and September and the lowest between March and April. These results are similar to that of another study [21], whereas Daugschies and Najdrowski [17] indicated an increase in spring in pastured calves in their first grazing season.

Rotavirus is the second most common pathogen detected in this study and a mixed infection with *Cryptosporidium* spp. was found to be in about half of the Rotavirus-positive samples. Also in former studies these two pathogens were the infectious agents, most often found in diarrhoeic young calves [50-52]. Bartels et al. [53] found a high rate of mixed infections of *Cryptosporidium* and Rotavirus in his study on young Dutch dairy calves. The investigation of different risk factors showed that one risk factor for *C. parvum* was the presence of one or more calves of the same age shedding Rotavirus. In that study the prevalence of both parasites was highest in 2-week old calves. In the study of Uhde et al. [52] Rotavirus was the infectious agent that was most often found in diarrhoeic calves, either alone or in mixed-infections with mostly *Cryptosporidium*. As discussed there, much indicates that Rotavirus is a primary pathogen causing diarrhoea in neonatal calves. That is why Rotavirus is thought to have a predominant role in the pathogenesis of neonatal calf diarrhoea in that study [52]. Which pathogen of the two most frequent – *Cryptosporidium* spp. or Rotavirus – has the predominant role in the present study remains unclear. In contrary to the high detection rates of Rotavirus and *Cryptosporidium* spp. low ones were found for *E. coli* and Coronavirus, what goes in line with the results of other European studies [51-53].

## Conclusions

This study shows that, in addition to *Eimeria* spp., *Giardia* spp. seems to play a contributing role of notable importance in diarrhoea in older calves, whereas *Cryptosporidium* spp. and Rotavirus are mostly relevant in young calves in Southern Germany.

## Competing interests

The authors declare that they have no competing interests.

## Authors’ contributions

JG carried out the examination by microscope and ELISA, analysed and interpreted the data and drafted the manuscript, DR performed the statistical analysis, KP and MS conceived of the study, participated in its design and conception and helped to draft the manuscript. All authors read and approved the final manuscript.
